# A UK survey of COVID‐19 related social support closures and their effects on older people, people with dementia, and carers

**DOI:** 10.1002/gps.5434

**Published:** 2020-09-25

**Authors:** Clarissa Giebel, Kathryn Lord, Claudia Cooper, Justine Shenton, Jacqueline Cannon, Daniel Pulford, Lisa Shaw, Anna Gaughan, Hilary Tetlow, Sarah Butchard, Stan Limbert, Steve Callaghan, Rosie Whittington, Carol Rogers, Aravind Komuravelli, Manoj Rajagopal, Ruth Eley, Caroline Watkins, Murna Downs, Siobhan Reilly, Kym Ward, Rhiannon Corcoran, Kate Bennett, Mark Gabbay

**Affiliations:** ^1^ Department of Primary Care and Mental Health University of Liverpool Liverpool UK; ^2^ NIHR ARC NWC Liverpool UK; ^3^ Centre for Applied Dementia Studies University of Bradford Bradford UK; ^4^ Division of Psychiatry University College London London UK; ^5^ Camden and Islington NHS Foundation Trust London UK; ^6^ Sefton Older People's Forum Sefton UK; ^7^ Lewy Body Society Wigan UK; ^8^ Lancashire Care NHS Foundation Trust Preston UK; ^9^ Department of Modern Languages and Cultures University of Liverpool Liverpool UK; ^10^ Together in Dementia Everyday (TIDE) Liverpool UK; ^11^ SURF Liverpool Liverpool UK; ^12^ Mersey Care NHS Foundation Trust Prescot UK; ^13^ EQE Health Liverpool UK; ^14^ Me2U Day Care Centre Liverpool UK; ^15^ National Museums Liverpool Liverpool UK; ^16^ North West Boroughs NHS Trust Warrington UK; ^17^ Liverpool Dementia Action Alliance Liverpool UK; ^18^ Faculty of Health and Wellbeing UCLAN Preston UK; ^19^ Department of Health Research Lancaster University Lancaster UK; ^20^ The Brain Charity Liverpool UK; ^21^ School of Psychology University of Liverpool Liverpool UK

**Keywords:** caring, COVID‐19, dementia, quality of life, social care, well‐being

## Abstract

**Objectives:**

The aim of this national survey was to explore the impact of COVID‐19 public health measures on access to social support services and the effects of closures of services on the mental well‐being of older people and those affected by dementia.

**Methods:**

A UK‐wide online and telephone survey was conducted with older adults, people with dementia, and carers between April and May 2020. The survey captured demographic and postcode data, social support service usage before and after COVID‐19 public health measures, current quality of life, depression, and anxiety. Multiple linear regression analysis was used to explore the relationship between social support service variations and anxiety and well‐being.

**Results:**

Five hundred and sixty‐nine participants completed the survey (61 people with dementia, 285 unpaid carers, and 223 older adults). Paired samples *t*‐tests and *X* ^2^‐tests showed that the mean hour of weekly social support service usage and the number of people having accessed various services was significantly reduced post COVID‐19. Multiple regression analyses showed that higher variations in social support service hours significantly predicted increased levels of anxiety in people with dementia and older adults, and lower levels of mental well‐being in unpaid carers and older adults.

**Conclusions:**

Being unable to access social support services due to COVID contributed to worse quality of life and anxiety in those affected by dementia and older adults across the UK. Social support services need to be enabled to continue providing support in adapted formats, especially in light of continued public health restrictions for the foreseeable future.

## 1 BACKGROUND

The first case of COVID‐19 was reported on New Year's Eve 2019 in Wuhan, China, and has since spread globally, putting many nations into lockdown. In the UK, a nationwide lockdown was imposed from the 23rd of March which lasted for over 12 weeks, not allowing people to go outside the home more than once a day and only for exercise or essential trips, such as grocery shopping or picking up medication. People aged 70+ years and those with underlying health conditions were not supposed to go outside at all and supposed to shield.

Across the globe, an estimated 50 million people are living with dementia,^1^ with many more caring for someone with dementia. As there is currently no disease modifying treatment, social care accounts for the great majority of dementia care costs. While residential care accounts for most of these costs, costs also include peer support groups, respite care, day care centres, and befriending services which provide vital activities and care for people living with dementia (PLWD) living in their own homes. Some of these social support services are provided by the voluntary sector, whilst others are provided by the governmental social care system, with some services being subject to financial support to access, whilst others have to be paid for by the individuals themselves. Social support services are linked to improved levels of well‐being^2^, ^3^ and quality of life for people with dementia.^4^ Older adults experience high rates of social isolation,^5^ and a third of UK older adults live alone.^6^ Social support services can provide an important life line to support them live well and engage socially. Greater social engagement has been linked to lower levels of loneliness and depression, and higher quality of life.^7^, ^8^

Even prior to the pandemic, access to dementia care varied with sociodemographic characteristics such as gender, area deprivation and ethnicity.^9^, ^10^ Many PLWD living in the community have an unmet need for social company,^11^ and there can be a concern that the impacts of COVID may widen existing inequalities. A recent qualitative investigation in the UK has reported that people affected by dementia have experienced distress due to reduced availability of social services during the pandemic, with feelings of loss of control, uncertainty, and higher levels of carer burden discussed.^12^ However, to date no quantitative data have evidenced the impact of these service closures on mental well‐being.

Preliminary findings indicate that mental health in the general UK population has declined since the onset of the pandemic and associated social changes.^13^ However, to our knowledge, this is the first study of mental health and wellbeing that has specifically recruited PLWD, their family carers, and older people; and the first to explore how availability of social services for PLWD has changed with the pandemic.

This cross‐sectional survey had two aims as follows: (1) to explore how social support service access by older adults and those affected by dementia changed in March 2020, at the time when COVID‐related public health measures were imposed; and (2) to explore, in people who were receiving social support services prior to the pandemic, the relationship between any change in service availability and mental well‐being, anxiety, and depression symptoms. Social support is defined as community‐based, non‐residential care. We hypothesised that participants would report receiving on average fewer hours of social support after COVID‐19 public health measures were instigated, relative to the weeks before the shutdown; and that receiving fewer hours of social support would be associated with worse well‐being, anxiety, and depression among people who are in receipt of care services.

**Key Points**

- Social support service usage for dementia has decreased significantly since the pandemic
- Higher variations in social support service usage compared to pre‐pandemic levels were associated with increased levels of anxiety in older adults and people with dementia
- Greater variation in social support service usage was also linked to lower levels of mental well‐being in unpaid carers and older adults
- The changes to social support services as a result of COVID‐19 public health restrictions are thus linked to wider well‐being of people with dementia, carers, and older adults

## 2 METHODS

### 2.1 Sampling and procedures

We recruited older adults aged 65+ years, people with dementia, and unpaid current and former carers of PLWD via: NIHR Join Dementia Research network, a UK‐based research registry for people with dementia, carers, and health volunteers interested in taking part in dementia research; social media; and third sector and social support service organisations, using mailing lists and newsletters, and direct approach by service providers via telephone or email. As potential participants were mostly approached via generic newsletters, social media announcements, and via existing and established networks with third sector organisations and service providers, methods also employed in the University College London COVID‐19 social study,^14^ no data on response rate is available. The survey could be completed either online or via phone with a research team member.

The decision to include former carers arose from discussions amongst the research team, which includes academics, clinicians, those affected by dementia, service providers, and third sector organisations. The rationale for this was that former carers often continue to access social support services, mostly peer support groups, after the PLWD's death in the UK.

PLWD were assumed to have capacity when taking part in the online survey. For those who completed the survey over the phone, researchers assessed mental capacity at the beginning according to the Mental Capacity Act.^15^

### 2.2 Survey variables

We asked participants whether they were a PLWD, a current carer, former carer, or aged 65+ years. Respondents were invited to tick all categories that applied. For the first three categories (PLWD, current carer and former carer), no participants ticked more than one of these responses. Where respondents ticked aged 65+ years and one of the other three categories, they were categorised as a PLWD, current or former carer. Thus, no participant in the older adult category had caring responsibilities.

#### 2.2.1 Demographic variables

These included age, gender, ethnicity, and for people with dementia, dementia subtype if known. Postcode data were collected to generate an Index of Multiple Deprivation (IMD) quintile. The IMD is an index of neighbourhood deprivation generating one deprivation score for income, employment, education, health, crime, barriers to housing and services, and living environment. Quintile ‘1’ indicates the least disadvantaged neighbourhoods, with quintile ‘5’ indicating the most disadvantaged neighbourhoods.

#### 2.2.2 Service receipt

We developed a brief questionnaire based on consultations with people affected by dementia and clinicians as part of a previous ongoing study. We asked all participants to state types and levels of social support service access, which were recorded in hours per week, including paid carers, support groups, social activities in the community, respite, day care centres, meal deliveries, befriending and accompanying services, and others, pre‐ (T1) and post‐ (T2) COVID‐19 (measured as in a typical week before and since the UK lockdown on the 23rd of March). From this data, we calculated a variable of variations in social support service usage, by calculating the difference in total weekly hours between T2 and T1.

#### 2.2.3 Psychological measures

Participants were asked to complete the following validated scales: The Short Warwick‐Edinburgh Mental Well‐Being Scale (SWEMWBS)^16^; this measures quality of life via seven items on a 5‐point Likert scale, with higher scores indicating higher levels of well‐being. The Generalised Anxiety Disorder 7 (GAD‐7)^17^ to measure anxiety in the past 2 weeks, with higher scores indicating higher levels of anxiety. The Personal Health Questionnaire 9 (PHQ‐9)^18^ measured levels of depression in the past 2 weeks. Higher scores indicate higher levels of depression. A cut off score of 10 on the GAD‐7 and the PHQ‐9 indicate moderate anxiety and depression, respectively.^19^

### 2.3 Data collection

The survey could be completed either online or via phone with a research team member who completed the online survey with details provided by the participant. The current data are from the baseline of an ongoing, longitudinal survey. Data were collected from 17th April to 15th May. We obtained ethical approval from the University of Liverpool prior to study begin (Ref: 7626).

### 2.4 Data analysis

We used SPSS 25 to analyse data. We used standard summary statistics to describe the data. A paired samples *t*‐test was used to compare the mean in hours of social support service usage before and since COVID. A Chi‐square test was used to compare the number of people having used no social support services at T1 and T2. To assess caseness of anxiety and depression, the cut off score for the GAD‐7 and the PHQ‐9 were employed to categorise participants into those with (‘1’) and without (‘0’) anxiety and depression. For each group (PLWD, unpaid carers and older adults), multiple regression analyses were employed where variations in hours of social support service usage were found to be significantly correlated with the continuous measures of SWEMWBS, GAD‐7, and PHQ‐9, via previously conducted bivariate correlation analysis. Dummy variables were created for IMD quintiles, with Quintile 5 (most disadvantaged) as the reference category. We included age, gender, living situation, years of education, and IMD quintiles in regression analyses as covariates, whilst checking for multi‐collinearity.

Participants with complete missing data on the PHQ‐9, GAD‐7, and SWEMWBS were removed from the total sample. There were no cases with only one or two items missing on the scale, but instead the entire questionnaire was incomplete.

## 3 RESULTS

### 3.1 Demographic characteristics

Six hundred sixty people participated in the survey, of which 25 were duplicates and 66 had large numbers of missing data, resulting in 569 participant cases included in this study (61 PLWD; 219 current carers; 66 former carers; 223 older adults). The majority of participants completed the survey online (93.5%).

The majority of participants were female (68%) and from a White ethnic background (97%) lived with other(s) (74%) and were retired (71%), with more participants living in less disadvantaged as opposed to more disadvantaged neighbourhoods, as measured by the IMD score. Most PLWD lived with and carers (had) cared for someone living with Alzheimer's disease dementia (41%), followed by mixed (23%) and vascular dementia (14%). PLWD and carers were on average 70 (+/−10) and 61 (+/−13) years of age, respectively, with the group of older adults interviewed who did not identify as living with dementia or being a carer for a person with dementia, having an average age of 72 (+/−6). Participants had on average 16 (+/−4) years of education. Table 1 shows all demographic characteristics of the sample by group.

**TABLE 1:** Demographic characteristics

	People with dementia (*n* = 61)	Current carers (*n* = 219)	Former carers (*n* = 66)	Older adults (*n* = 223)	Total sample (*n* = 569)
*N*(%)					
Gender					
Female	27 (44.3)	168 (77.1)	55 (83.3)	137 (61.7)	387 (68.3)
Male	34 (55.7)	50 (22.9)	11 (16.7)	85 (38.3)	180 (31.7)
Ethnicity					
White	58 (95.1)	211 (96.3)	65 (98.5)	216 (98.2)	550 (97.2)
BAME	2 (3.3)	7 (3.3)	0	3 (1.4)	12 (2.1)
Not wish to say	1 (1.6)	1 (0.5)	1 (1.5)	1 (0.5)	4 (0.7)
IMD quintile					
1	12 (23.1)	54 (32.1)	10 (19.2)	61 (33.5)	137 (30.2)
2	16 (30.8)	50 (29.8)	20 (38.5)	44 (24.2)	130 (28.6)
3	10 (19.2)	32 (19.0)	14 (26.9)	37 (20.3)	93 (20.5)
4	10 (19.2)	14 (8.3)	5 (9.6)	26 (14.3)	55 (12.1)
5	4 (7.7)	18 (10.7)	3 (5.8)	14 (7.7)	39 (8.6)
Living situation					
Living alone	13 (21.3)	33 (15.1)	17 (26.2)	79 (35.6)	142 (25.1)
Living with someone	48 (78.7)	185 (84.9)	48 (73.8)	143 (64.4)	424 (74.9)
Employment status					
Full‐time	2 (3.3)	42 (19.3)	11 (16.9)	7 (3.2)	62 (11.0)
Part‐time	2 (3.3)	43 (19.3)	4 (6.2)	19 (8.6)	67 (11.9)
Unemployed	1 (1.7)	24 (11.0)	2 (3.1)	2 (0.9)	29 (5.1)
Retired	53 (88.3)	108 (49.5)	46 (70.8)	193 (87.3)	400 (70.9)
Not wish to say	2 (3.3)	2 (0.9)	2 (3.1)	‐	6 (1.1)
Type of dementia					
Alzheimer's	20 (32.8)	100 (46.5)	6 (23.1)	‐	127 (41.4)
Mixed	13 (21.3)	49 (22.8)	7 (26.9)	‐	69 (22.5)
Vascular	11 (18.0)	27 (12.6)	4 (15.4)	‐	43 (14.0)
Other	17 (27.9)	39 (18.1)	9 (34.5)	‐	68 (22.2)
Mean (SD), (range)					
Age	70 (+/−10), (45–88)	61 (+/−13), (23–89)	64 (+/−14), (22–95)	72 (+/−6), (65–90)	67 (+/−12), (22–95)
Years of education	15 (+/−4), (4–25)	16 (+/−4), (6–28)	17 (+/−4), (10–29)	17 (+/−4), (7–25)	16 (+/−4), (4–29)
Median (range)					
GAD‐7 total (possible range 0–21)	7 (0–20)	6 (0–21)	4 (0–18)	1 (0–18)	4 (0–21)
PHQ‐9 total (possible range 0–27)	9 (0–24)	5 (0–21)	4 (0–18)	2 (0–19)	4 (0–24)
SWEMWBS total (possible range 0–35)	22 (7–35)	24 (11–35)	25 (12–35)	28 (11–35)	26 (7–35)

*Notes*: Higher scores on the GAD‐7, PHQ‐9, and the SWEMWBS indicate higher levels of anxiety, depression and well‐being, respectively. IMD Quintile 1 indicates the least disadvantaged neighbourhoods and Quintile five the most disadvantaged neighbourhoods.

Abbreviations: BAME, black and minority ethnic; GAD‐7, General Anxiety Disorder; PHQ‐9, Personal Health Questionnaire; SWEMWBS, Short Warwick and Edinburgh Mental Well‐Being Scale.

### 3.2 Social support service usage at T1 and T2

Mean weekly hours of accessing social support services was 12.0 (SD = 28.5; Skewness = 4.5; Kurtosis = 21.0) at T1 and 6.6 (SD = 29.5; Skewness = 5.1; Kurtosis = 24.9) at T2 (paired *t*(390) = 4.894, *p* < .001). Two hundred and fifty‐one participants reported having received >0 h at T1 (31 PLWD, 156 current carers, 20 former carers, 44 older adults). Of those, the variation in mean number of hours between T1 and T2 was 9.0 (+/−23.4), ranging from −162 to +168, with some participants experiencing fewer hours at T2, and others more hours.^1^

**FIGURE 1:**
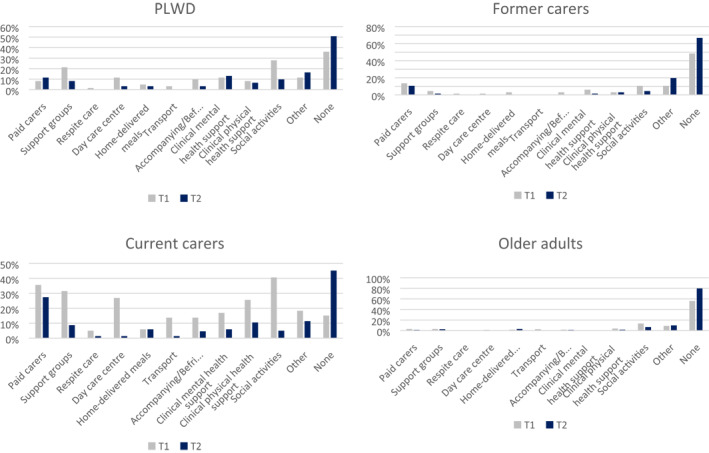
Social support service usage before and since COVID‐19 lockdown by group. T1 = Before COVID‐19 lockdown; T2 = Since COVID‐19 lockdown. Bar charts represent the proportion of participants within each group who reported having accessed individual social support services pre and since COVID

Figure 1 shows social support service usage at T1 and T2 by group. Engaging in social activities in the community, such as singing and dancing groups, was accessed the most at T1, followed by accessing peer support groups and receiving paid carers in the home, as well as day care centres. Few carers accessed respite care. PLWD and current carers accessed most services both at T1 and T2. Out of all types of services, paid carer support was the least affected, with access to all services having declined since the outbreak (except paid carer access for PLWD), and the number of people having accessed no support services having risen across all groups (in the total sample from 212 [37.3%] to 352 [61.9%]). Chi‐square test showed that the number of participants receiving no social support at T2 was significantly larger than at T1 (*X* ^2^(1569) = 117.994, *p* < .001).

Table 2 describes the types and amount of social support service for the total sample. A significant number of people showed a reduction in having accessed no form of social support service between T1 and T2.

**TABLE 2:** Social support service usage before and since COVID‐19 lockdown

Type of social support	T1	T2
Paid carers	99 (17.4%)	77 (13.5%)
Support groups	92 (16.2%)	31 (5.4%)
Respite care	14 (2.5%)	3 (0.5%)
Day care centre	70 (12.3%)	5 (0.9%)
Home‐delivered meals	22 (3.9%)	22 (3.9%)
Transport	38 (6.7%)	3 (0.5%)
Accompanying/Befriending	42 (7.4%)	15 (2.6%)
Clinical mental health support	49 (8.6%)	22 (3.9%)
Clinical physical health support	72 (12.7%)	33 (5.8%)
Social activities	143 (25.1%)	35 (6.2%)
Other	74 (13.0%)	70 (12.3%)
None	212 (37.3%)	352 (61.9%)

*Note*: Table shows number of participants (%) of the total sample (n = 617) who accessed various types of social support services at T1 (pre COVID‐19) and at T2 (since lockdown).

### 3.3 Mental well‐being

**FIGURE 2:**
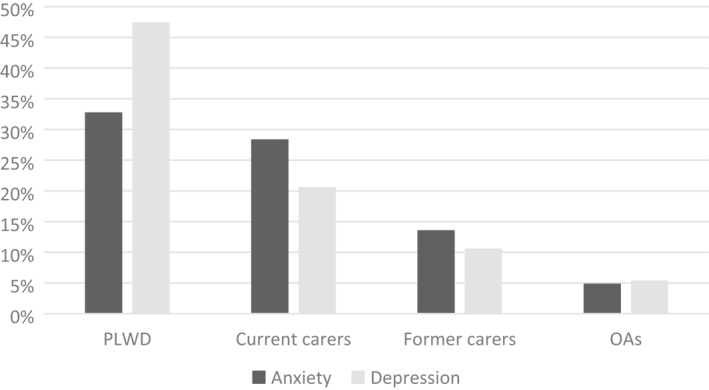
Prevalence of anxiety and depression by group. The diagram shows the proportion of PLWD, carers, and older adults who scored 10 or above on the GAD‐7 or PHQ‐9 for anxiety and depression, respectively. OAs, Older adults; PLWD, People living with dementia

The group with the highest proportion scoring above the cut off for both anxiety (33%) and depression (48%) were those living with dementia. In contrast, far fewer older adults achieved caseness (5% anxiety and 5% depression). Amongst carers, proportions of anxiety and depression were higher amongst current (28%, 20%) than former carers (14%, 11%). Figure 2 summarises these findings.

### 3.4 Multiple regression analysis on changes in social support service hours and well‐being and anxiety

Of those having received >0 h at T1 (*n* = 251), we combined current and former carers into one single carers group (*n* = 179), to increase the power of this group for the regression model.

Bivariate correlation analysis prior to the regression modelling shows that for PLWD, variation in social support hours was significantly related only to higher GAD‐7 scores (*p* < 0.05), not the SWEMWBS (*p* = 0.392) or PHQ‐9 (*p* = 0.862). For carers, variation in social support hours was significantly related to the reduced scores on the SWEMWBS (*p* < 0.01), not the GAD‐7 (*p* = 0.777) or the PHQ‐9 (*p* = 0.475). For older adults, hours were significantly related to reduced scores on the SWEMWBS (*p* < 0.05) and higher scores on the GAD‐7 (*p* < 0.05), not the PHQ‐9 (*p* = 0.155).

Four multiple regression analyses were conducted (see Table 3 for further details). Variation in social support service hours were significantly related with anxiety in PLWD (*p* < 0.05), mental well‐being in carers (*p* < 0.05), and mental well‐being and anxiety in older adults (*p* < 0.05; *p* < 0.05), all of whom had received at least some weekly hours pre COVID. When running the multiple regression analysis for carers with current carers only, results were comparable to the model with both former and current carers merged, but variation in hours of social support service usage failed to achieve statistical significance (*p* > 0.05). Higher levels of variation in hours of support were related to higher levels of anxiety and lower levels of well‐being. Other factors such as age, gender, years of education, living situation (alone/with others), and IMD quintiles were not found to be significantly associated.

**TABLE 3:** Multiple linear regression analyses on predictors of mental well‐being by group

	Beta	Standard error	*p*‐value	Standardised beta	95% confidence interval
GROUP 1: People living with dementia (*N* = 30)					
GAD‐7					
Age	−0.049	0.110	0.659	−0.096	−0.275 to 0.177
Gender	0.518	2.329	0.826	0.048	−4.279 to 5.316
Years of education	0.377	0.229	0.113	0.295	−0.095 to 0.848
Living situation	−2.684	2.789	0.345	−0.197	−8.428 to 3.060
Variation in social support service hours^a^	0.107	0.040	**0.012**	0.465	0.025 to 0.188
*R* ^2^ = 0.305, *F*(5, 25) = 2.196, *p* = 0.087					
GROUP 2: Carers (*N* = 172)					
SWEMWBS					
Age	0.081	0.032	**0.014**	0.199	0.017 to 0.145
Gender	0.424	1.016	0.677	0.033	−1.581 to 2.428
Variation in social support service hours	−0.034	0.015	**0.022**	−0.171	−0.064 to −0.005
*R* ^2^ = 0.084, *F*(3, 169) = 5.172, *p* < 0.01					
GROUP 3: Older adults (*N* = 32)					
SWEMWBS					
Age	−0.265	0.198	0.194	−0.302	−0.674 to 0.145
Years of education	0.240	0.381	0.535	0.129	−0.547 to 1.026
Living situation	2.101	2.447	0.399	0.163	−2.950 to 7.152
Variation in social support service hours	−0.452	0.167	**0.012**	−0.471	−0.798 to −0.107
IMD quintile 1^b^	−3.069	6.328	0.632	−0.213	−16.130 to 9.992
IMD quintile 2^b^	−1.200	6.236	0.849	−0.088	−14.072 to 11.671
IMD quintile 3^b^	−4.837	6.449	0.461	−0.308	−18.148 to 8.474
IMD quintile 4^b^	2.398	6.876	0.730	0.134	−11.793 to 16.590
*R* ^2^ = 0.409, *F*(8, 24) = 2.077, *p* = 0.080					
GAD‐7					
Age	−0.039	0.139	0.778	−0.062	−0.322 to 0.243
Gender	−0.574	1.591	0.721	−0.059	−3.816 to 2.667
Years of education	0.044	0.267	0.870	0.034	−0.500 to 0.587
Living situation	−0.402	1.662	0.810	−0.045	−3.787 to 2.982
Variation in social support service hours	0.297	0.120	**0.019**	0.415	0.052 to 0.542
*R* ^2^ = 0.163, *F*(5, 32) = 1.249, *p* = 0.310					

*Notes*: Levels of anxiety and mental well‐being are measured at one point in time. Bold highlighted *p*‐values indicate statistical significance.

Abbreviations: GAD‐7, Generalised Anxiety Disorder seven; IMD, Index of Multiple Deprivation; SWEMWBS, Short Warwick and Edinburgh Mental Well‐Being Scale.

^a^ Variations in social support service hours = Weekly total hours at T2–T1.

^b^ IMD quintile 5 (most disadvantaged) is the reference category.

## 4 DISCUSSION

Our study is the first to quantify how the pandemic has impacted social support service availability, and to explore the impact of this on the lives of people affected by dementia as well as older adults across the UK. For those who had received social support services pre COVID, reductions in weekly social support service hours were significantly associated with reduced levels of well‐being in carers and older adults and anxiety in PLWD and older adults. At this stage, we did not demonstrate a relationship with depression.

As a result of COVID‐related public health restrictions, nearly all forms of social support services, including day care centres, peer support groups, and social activities in the community, have had to stop face‐to‐face service provision at least temporarily. Among our sample, over a third received no support at all pre COVID, while the majority of people were found to not receive any form of social support since COVID. Social support services form a crucial part of post‐diagnostic dementia care and meeting the needs of older adults, many of whom experience high levels of loneliness.^20^

Among people affected by dementia and older adults, being unable to access support services since COVID was significantly related to reductions in well‐being and increases in anxiety. For PLWD, being unable to visit their usual support services predicted higher levels of anxiety. Our complementary qualitative research has found that both PLWD and unpaid carers experienced high levels of uncertainty about when and how services will resume.^12^ In the study, many PLWD were reported to not comprehend the public health restrictions of social distancing and lockdown, and whilst PLWD in the present study had mental capacity and completed the survey themselves, there may possibly also be an underlying issue of not comprehending the restrictions fully.

For carers, being unable to access previously utilised activities significantly predicted their well‐being. Unpaid carers provide a large proportion of dementia care, worth over £13 billion each year in the UK.^21^ While carers might not always acknowledge themselves how much care they provide, ranging from preparing a hot meal to dressing the PLWD or supporting them to use the toilet, carers can become increasingly burdened as the dementia progresses and symptoms advance with PLWD requiring more support.^22^ Accessing respite care and having some time to themselves whilst the PLWD is attending a day care centre is therefore crucial to support the carer. With COVID and restrictions in place, these opportunities have suddenly been taken away from carers, leaving them likely at a loss of how to adapt the care required for the PLWD. Carers are thus likely to have to pick up increased care hours, or merely get no respite from caring for someone 24/7, which might explain the significant association with their well‐being as evidenced in this study. However, former carers have also been affected, as the desire and need to access peer support after their relative has passed away stays in place for many carers. Therefore, findings clearly highlight the need for both current and former carers to continue accessing social support.

COVID‐related closures were also significantly related to lower mental well‐being and higher levels of anxiety in older adults. In our sample, older adults had the highest proportion of those living alone (34%). They mostly accessed social activities in the community pre COVID, which include for example arts groups or other hobbies. These are important for enabling social inclusion and social interactions, and the link between social engagement and well‐being is well established in the literature.^23^ Previous research has shown a linear relationship between anxiety and social isolation,^24^ which is likely to explain our findings. However, the global pandemic in itself and the stress associated with this is likely to be related to high levels of anxiety also.

There are numerous strengths to this study, with a relatively large sample, with responses across the UK, and relatively high proportions of rare dementias. Some of the limitations relate to the sample demographics, as people were highly educated, predominantly from a White ethnic background and living in more affluent areas. This might also be linked to the fact that the majority of participants participated in the survey online and not over the phone, and thus not capturing people from more disadvantaged backgrounds who might not have Internet access. Whilst we approached black and minority ethnic (BAME) dementia support groups to share the survey link and study information, the majority of participants were from a White ethnic background. This somewhat limits the representativeness of our findings, as a recent report estimated that around 7% of PLWD in England and Wales are from a BAME.^25^ Future surveys need to investigate these experiences particularly in people from lower socio‐economic backgrounds and those from minority ethnic groups, some of the worst affected groups from COVID‐19.^26^ Considering the multi‐pronged recruitment and sampling strategy, it was not possible to obtain a response rate. However, other recently published COVID‐19 surveys using similar sampling strategies^14^ were equally not able to obtain a response rate, and thus our study adheres to other ongoing COVID‐19 survey methodologies. As part of this, while convenience sampling helped to recruit as large a sample as possible, it can be limiting as we did not purposefully recruit for people from minority groups for example, such as people from BAME. There may be problems with recall bias, particularly amongst those living with dementia. Some telephone interviewers noted that participants sometimes had to be asked in more depth about accessed support services, as they would otherwise not have reported certain types of support. This could have led to a general under‐reporting of some social support service usage in our study. However, we are not aware of any currently routinely collected data on social service and social care provision. In addition, as part of this survey it was not possible to collect data on PLWD's dementia severity, such as via established measures (i.e., Clinical Dementia Rating Scale) or purely cognitive performance on measures (i.e., Mini Mental State Examination). Therefore, we are unable to state precisely how far advanced the condition was for PLWD. However, all PLWD are very likely to be in the very early to mild stages of the condition, as otherwise they would have been unable to complete the survey.

## 5 CONCLUSIONS

Social support services are vital to support the mental well‐being of older adults and people affected by dementia. Our research demonstrated a link between COVID‐related service loss and detrimental impacts on these vulnerable groups. With public health restrictions such as social distancing likely to stay in place for some time to come, it is important to find ways to adapt these services to seeking alternative ways to re‐provide support to meet the needs of those requiring social support. This can in some way achieved by providing remote support services, which is very slowly being trialled with very little evidence to date though (Cheung & Peri^27^; Goodman‐Casanova et al.^28^). However, remote service support does not substitute face‐to‐face support, so that a right mix of different formats needs to be provided, in a safe environment, to enable older adults and people affected by dementia access support services throughout the pandemic. There is evidence that social support reduces the risk of care home admissions and unplanned hospital admissions. Therefore, it is important to act now and enable pre‐pandemic levels of social support, if not better, as otherwise health care and social care services will be overburdened with increased rates of cost‐intensive care home admissions and healthcare visits.

## CONFLICT OF INTEREST

We declare no competing interests.

## AUTHOR CONTRIBUTIONS

Clarissa Giebel conceptualised the study. Clarissa Giebel, Claudia Cooper, and Mark Gabbay developed the study analysis in consultation with the team. Daniel Pulford managed recruitment. Clarissa Giebel performed data analysis and draughted the manuscript. All co‐authors interpreted the findings jointly, provided feedback on drafts of the manuscript, and approved the final draft.

## Data Availability

The data that support the findings of this study are available from the corresponding author upon reasonable request.

## ENDNOTE

^1^
One case reported 168 weekly hours of variations in social support services.
